# Cost-Utility Analysis of Screening for Diabetic Retinopathy in China

**DOI:** 10.34133/2022/9832185

**Published:** 2022-03-12

**Authors:** Yue Zhang, Weiling Bai, Ruyue Li, Yifan Du, Runzhou Sun, Tao Li, Hong Kang, Ziwei Yang, Jianjun Tang, Ningli Wang, Hanruo Liu

**Affiliations:** ^1^Beijing Tongren Eye Center, Beijing Key Laboratory of Ophthalmology and Visual Sciences, Beijing Tongren Hospital, Capital Medical University, Beijing Institute of Ophthalmology, Beijing, China; ^2^College of Computer Science, Nankai University, Tianjin, China; ^3^School of Agricultural Economics and Rural Development, Renmin University of China, Beijing, China; ^4^National Institute of Health Data Science at Peking University, Beijing, China; ^5^Beijing Institute of Ophthalmology, Beijing Tongren Hospital, Capital Medical University, Beijing Ophthalmology & Visual Science Key Lab, Beijing, China; ^6^School of Information and Electronics, Beijing Institute of Technology, Beijing, China

## Abstract

*Background*. Diabetic retinopathy (DR) has been primarily indicated to cause vision impairment and blindness, while no studies have focused on the cost-utility of telemedicine-based and community screening programs for DR in China, especially in rural and urban areas, respectively.*Methods*. We developed a Markov model to calculate the cost-utility of screening programs for DR in DM patients in rural and urban settings from the societal perspective. The incremental cost-utility ratio (ICUR) was calculated for the assessment.*Results*. In the rural setting, the community screening program obtained 1 QALY with a cost of $4179 (95% CI 3859 to 5343), and the telemedicine screening program had an ICUR of $2323 (95% CI 1023 to 3903) compared with no screening, both of which satisfied the criterion of a significantly cost-effective health intervention. Likewise, community screening programs in urban areas generated an ICUR of $3812 (95% CI 2906 to 4167) per QALY gained, with telemedicine screening at an ICUR of $2437 (95% CI 1242 to 3520) compared with no screening, and both were also cost-effective. By further comparison, compared to community screening programs, telemedicine screening yielded an ICUR of 1212 (95% CI 896 to 1590) per incremental QALY gained in rural setting and 1141 (95% CI 859 to 1403) in urban setting, which both meet the criterion for a significantly cost-effective health intervention.*Conclusions*. Both telemedicine and community screening for DR in rural and urban settings were cost-effective in China, and telemedicine screening programs were more cost-effective.

## 1. Introduction

Diabetic retinopathy (DR) has been primarily indicated to cause vision impairment and blindness, and the risk of developing DR is high in diabetes mellitus (DM) patients [1]. In 2019, a national cross-sectional survey in China reported 116 million people with diabetes [2], and China was estimated to have the largest population of adult patients with DM worldwide [3– 5]. DR can be mainly divided into no apparent DR; mild, moderate, and severe nonproliferative diabetic retinopathy (NPDR); and proliferative diabetic retinopathy (PDR) [6]. Severe vision impairment largely occurs with PDR and diabetic macular edema (DME). Regular DR screening should be conducted to prevent DR and control the progression from mild DR to severe vision impairment [7].

Screening and timely treatment prior to symptoms can significantly reduce the severe visual loss and economic burden from vision-threatening DR and DME [8– 10]. Several studies have recognized that patients with DR can benefit from regular screening and appropriate referrals to ophthalmology health care [11– 14]. According to a series of European screening studies, free DR screening services have reportedly reduced the prevalence of blindness by more than two-thirds [15, 16].

The Chinese guidelines for the prevention and treatment of DR recommend that DM patients receive an annual fundus enlargement examination, and timely interventions help reduce the risk of loss of vision in PDR and DME patients [17, 18]. However, as suggested from existing surveys, nearly two-thirds of DM patients have not undergone an examination for more than 1 year [19, 20]. Currently, there is no comprehensive national screening and prevention system for DR in China [21]. Moreover, according to previous research, there is a higher prevalence of DR in rural China than that in those living in urban areas [22]. Patients’ awareness of DR in rural areas is lower than urban areas due to lower economic development level and restricted primary health resources, which might delay diagnosis and nonoptimal control of DR in rural China. Telemedicine screening programs can reduce the burden of conventional clinical examination and improve access in rural areas. As indicated by existing studies, screening programs based on telemedicine platforms are cost-effective compared with conventional screening, including Singapore, the United States, India, and Brazil [23, 24]. However, no studies have focused on the cost-utility of teleophthalmology in China, especially in rural and urban areas, respectively [23].

To fill the identified gaps, this study was aimed at (1) calculating the cost-utility of screening programs compared with no screening programs that target DM patients in rural and urban areas, (2) comparing the cost-utility of telemedicine-based and community screening programs, and (3) providing advice for screening intervals under different settings.

## 2. Methods

### 2.1. Model Design

**Figure 1:**
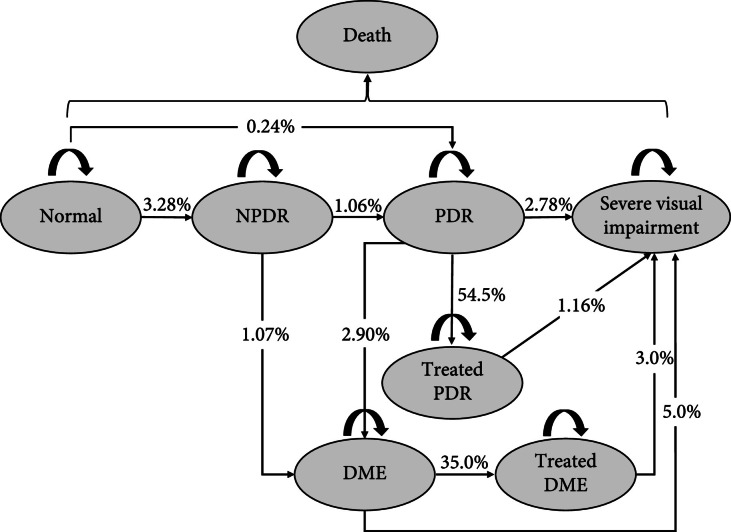
Markov model showing possible transitions across health states. NPDR = nonproliferative diabetic retinopathy; PDR = proliferative diabetic retinopathy; DME = diabetic retinopathy edema.

We used TreeAge Pro (TreeAge Software; Williamstown, MA, USA) to build a Markov model from scratch to estimate the cost-utility of screening programs for DR in DM patients in China, from the societal perspective. The study population was a hypothetical cohort of patients with DM not previously screened for DR and with a mean age of 50 years, analyzed with 30 Markov cycles in total. Simulated patients were allocated into one of six Markov health states: no DR, NPDR, PDR, DME, severe visual impairment (bilateral best-corrected visual acuity <6/60), and death (Figure 1). 

In each Markov cycle, the transitions between health states were as follows: (a) patients with no DR may remain or progress to NPDR or PDR; (b) NPDR patients may progress into PDR or DME or remain as NPDR; (c) PDR patients may remain or progress to DME or severe visual impairment; (d) DME patients may remain or progress to severe visual impairment. We assumed that NPDR patients received no treatment and needed an annual follow-up examination, and the patients would remain or progress to severe visual impairment after treatment.

### 2.2. Model Inputs

Data on the prevalence of each diabetic retinopathy type incorporated into the model are shown in Table 1, which is derived from a recent meta-analysis and epidemiological studies [22, 25, 26]. Transition probabilities in the natural state were calculated from studies in Asian nations, and transition probabilities between states after interventions were collected from published reviews (Table 1) [27– 33]. In studies on multiyear DR incidence reported, one-year incidence was calculated using the formula $r=-\log\left(1-p\right)/t$, where r is the one-year incidence and p is the cumulative incidence over length of interval t. 

**Table 1:** Markov model clinical parameter estimates.

Parameter	Base-case value	Range (95% CI)	Source
Prevalence			
NPDR in rural DM patients	0.256	0.181-0.348	[22, 25]
PDR in rural DM patients	0.016	0.003-0.071	[22, 25]
DME in rural DM patients	0.035	0.032-0.039	[25, 26]
NPDR in urban DM patients	0.149	0.106-0.204	[22, 25]
PDR in urban DM patients	0.011	0.005-0.023	[22, 25]
DME in urban DM patients	0.026	0.023-0.029	[25, 26]
Annual transition probabilities			
Normal to NPDR	0.0328	0.0184-0.0578	[27– 33]
Normal to PDR	0.0024	0.0009-0.0063	[27– 33]
NPDR to PDR	0.0106	0.0040-0.0278	[27– 33]
NPDR to DME	0.0107	0.0096-0.0118	[27– 33]
PDR to DME	0.0290	0.0261-0.0319	[27– 33]
PDR to severe visual impairment	0.0278	0.0100-0.0744	[27– 33]
DME to severe visual impairment	0.0500	0.0450-0.0550	[27– 33]
Treated PDR to severe visual impairment	0.0116	0.0005-0.0268	[31]
Treated DME to severe visual impairment	0.0300	0.0270-0.0330	[36]
Utility scores			
Normal	0.95	0.92-0.99	[37, 38]
NPDR	0.79	0.71-0.87	[37, 38]
PDR	0.70	0.63-0.77	[37, 38]
DME	0.70	0.63-0.77	[37, 38]
Severe visual impairment	0.55	0.50-0.61	[37, 38]
Characteristics of community screening			
Normal called NPDR	0.05	0.04-0.06	[19, 34, 39]
Normal called normal	0.95	0.86-1.00	[19, 34, 39]
NPDR called normal	0.22	0.20-0.24	[19, 34, 39]
PDR called NPDR	0.03	0.02-0.04	[19, 34, 39]
PDR called normal	0.02	0.01-0.03	[19, 34, 39]
Sensitivity of DME	0.82	0.74-0.90	[19, 34, 39]
Specificity of DME	0.79	0.71-0.87	[19, 34, 39]
Characteristics of telemedicine screening			
Normal called NPDR	0.04	0.03-0.05	[34, 37, 38]
Normal called normal	0.96	0.86-1.00	[34, 37, 38]
NPDR called normal	0.42	0.38-0.46	[34, 37, 38]
PDR called NPDR	0.19	0.17-0.21	[34, 37, 38]
PDR called normal	0.02	0.01-0.03	[34, 37, 38]
Sensitivity of DME	0.80	0.72-0.88	[34, 37, 38]
Specificity of DME	0.95	0.86-1.00	[34, 37, 38]
Mortality multipliers			
Diabetes	1.97	—	[39]
Severe visual impairment	3.9	—	[40]

DM = diabetes mellitus; DR = diabetic retinopathy; NPDR = nonproliferative diabetic retinopathy; PDR = proliferative diabetic retinopathy; DME = diabetic macular edema.

By synthesizing the data in existing studies in China and our unpublished data from the Handan ophthalmologic screening program, screening sensitivity and specificity, compliance with screening programs, and treatments at the community level and by telemedicine in rural and urban areas were determined [34– 36]. Utility was expressed in terms of quality-adjusted life years (QALYs) gained. The utility value was adopted to evaluate the quality of life related to each health stage (Table 1) [37, 38]. We referred to utility values from studies done in other Asian countries (such as India and Singapore). The values mentioned have been converted to QALYs as the ultimate unit of utility for the cost-utility analysis. Mortality risks are the age-specific mortality risks inflated by the risk ratios for diabetes (1.97), and for severe visual impairment (3.9) [39, 40], and assumed not to vary with age or DR stage (Table 1). 

Costs and health-state utilities were discounted at 3.5% per year in the base-case analysis. The WHO has given recommendations on the economic evaluation of disability-adjusted life years (DALYs) as the output indicator. A health intervention is defined as cost-effective if it costs less than three times the per capita gross domestic product (GDP) of a given country and significantly cost-effective if it costs less than per capita GDP [41]. Given the conceptual similarities between QALYs and DALYs, when QALYs act as the output indicator, the recommendations made by the WHO can also be referenced. In China, it was calculated as $7,000 and $12,000, respectively, from the overall per capita national GDP ($10137.98), urbanization rate (0.61), and urban-rural ratio (2.64) of per capita disposable income [42]. 

### 2.3. Screening and Intervention Costs

Under the societal perspective, costs consisted of direct costs (medical and nonmedical) and indirect costs. Direct medical costs include cost for DR screening and cost for follow-up and treatment. Direct nonmedical costs included transportation costs associated with visits to the hospitals. Indirect costs included the loss of monetary value due to visual impairment (e.g., severe visual impairment).

Screening costs in the community were calculated based on the Handan ophthalmologic screening program [25, 43]. Screening cost based on teleophthalmology was collected from the Center of Teleophthalmology Consultation in the Beijing Tongren Eye Center. In order to annualize the cost of a capital item, we could divide the replacement cost of capital by the annualization factor given by $\left[{\left(1+r\right)}^{n}-1\right]/\left[r{\left(1+r\right)}^{n}\right]$, where r is the discount (interest) rate and n is the useful life of the capital item [44]. Our cost standards were similar to those of most screening programs and tertiary hospitals in China. All figures were recorded in Chinese yuan and converted to US dollars at an exchange rate of 6.9762 yuan per dollar, according to RMB exchange rate published by the Monetary Policy Department of the People's Bank of China on 2020. The number of people screened each year was assumed to be 20,000, referring to the actual situation of the Handan ophthalmologic screening program and the Center of Teleophthalmology Consultation in Beijing Tongren Eye Center [26]. After the calculation, the total cost per person for community DR screening was $2.44, which is higher than the teleophthalmology cost of $1.84. 

Participants deemed positive through community screening examinations or teleophthalmology advice were referred to the hospital for full ophthalmologic examination (screening and referral pathway presented in Supplementary 1). Patients with suspected DR underwent several examinations. Regular follow-up observation was recommended for patients diagnosed with NPDR. PDR patients received scatter or panretinal photocoagulation in accordance with the specific condition. DME patients received intravitreal antivascular endothelial growth factor (VEGF) therapy [45]. Considering the complexity of the surgical cost (e.g., vitrectomy), it was not included in our model. All check and intervention costs used here were the amounts charged for medical care in the Beijing Tongren Hospital. The total cost for the initial year of treatment for patients with severe visual impairment was assumed to be $8800, and $3600 was incurred in subsequent years until death in the model, as suggested in an existing study [46]. Details of the compositions of all costs are listed in Table 2. 

Table 2Cost of screening program, hospital ophthalmologic examination, and treatment.

**(a):** 

Community screening costs (Handan ophthalmologic screening program)			
Cost items	Units	Unit cost ($)	Annualized cost ($)
Equipments			
Dilated pupils using a digital nonmydriatic retinal camera (Canon CR-DGi with a 20D SLR back; Canon, Tokyo, Japan)	1	24,000	5,321.51
Visual acuity measurement (logarithmic visual acuity chart)	1	250	55.43
Laptop	1	500	110.86
Medical staff	6	6,500	39,000
Other expenses			
Production of posters, leaflets, and other loose expenses	—	—	700
Casual supporting personnel distributing leaflets	—	—	640
Transportation cost	—	—	3,000
Number of people screened per year (assumption)	20,000		
Cost per person	2.44

**(b):** 

Telemedicine screening costs (Center of Teleophthalmology Consultation in Beijing Tongren Eye Center)			
Cost items	Units	Unit cost ($)	Annualized cost ($)
Equipment			
Computer software and peripherals	1	9,000	1,995.57
Installation	1	5,000	1,108.65
Other expenses			
Contract cost to other organizations	1	1,700	1,700
Medical staff for specialists	10	2,500	25,000
Medical staff for technicians	2	2,500	5,000
Maintenance cost	1	2,000	2,000
Number of people screened per year (assumption)	20,000		
Cost per person	1.84

**(c):** 

Hospital ophthalmologic examination and treatment cost (Beijing Tongren Hospital)	
Cost items	Cost ($)
Medical service	8.48
Fundus photography	4.74
Optical coherence tomography	13.85
Visual acuity and noncontact intraocular pressure measurement	0.78
Transportation and accommodation	63.76
Internal medical examination	8.39
Cost in total per person	100
Treatment costs (scatter or panretinal photocoagulation)	137.60
Treatment costs (intravitreal antivascular endothelial growth factor therapy)	1741.46
Maintain costs (follow-up observation)	122.32

^*^Annualized cost for fix assets (i.e., equipment) was calculated with the discounting rate of 3.5% and annualizing factor of 4.51, by assuming a life span 5 years. Salary of medical staff refers to the annual gross salary.

### 2.4. Cost-Utility Analysis

The main outcomes were incremental cost-utility ratios (ICURs) for telemedicine versus community screening programs under rural and urban settings. ICUR was calculated as the difference in total costs divided by the difference in total QALYs between screened and unscreened cohorts. Negative ICURs, which were considered dominating, show that screening results in fewer costs while increasing QALYs compared to no screening.

We calculated ICURs in one-off screening in rural and urban settings, to compare community and telemedicine screening methods to no screening, respectively. We calculated ICURs in one-off screening and different screening intervals by both screening methods in rural and urban settings. Strategies with shorter screening intervals generated higher costs and higher utility. If the ICURs were lower than the threshold of three times per capita GDP, screening intervals were recommended.

A half-cycle correction was employed for costs and benefits. The reporting of methods and results complied with the Consolidated Health Economic Evaluation Reporting Standards (Supplementary 6). 

### 2.5. Sensitivity Analysis

We conducted both one-way deterministic and simulated probabilistic sensitivity analyses. For the one-way deterministic sensitivity analysis, the parameters in our model were allowed to change within relatively large ranges (±10% of the mean for probabilities, utilities, and testing accuracy and either 20% or 50% of the mean for costs) (Supplementary 2). For the probabilistic sensitivity analysis, a beta distribution was applied to prevalence, utilities, and transition probabilities, and a gamma distribution was applied to cost parameters. We used a percentile-based nonparametric bootstrap method to calculate the 95% CIs for the ICURs. The results are presented as cost-effectiveness acceptability curves to show the probability of each screening model for a given cost-utility threshold. 

## 3. Results

As indicated by the cost-utility analysis, both telemedicine and community screening for DR under rural and urban settings dominated no screening. Under the rural setting, 1 QALY was gained through community screening at a cost of $4179 (95% CI 3859 to 5343) and through telemedicine screening at an ICUR of $2323 (95% CI 1023 to 3903), both of which satisfied the criterion for a significantly cost-effective health intervention. Likewise, per QALY gained, community screening in urban areas yielded an ICUR of $3812 (95% CI 2906 to 4167), and telemedicine screening delivered an ICUR of $2437 (95% CI 1242 to 3520), which were also cost-effective (Table 3).

**Table 3:** Summary of ICUR data of telemedicine and community screening compared with no screening in rural and urban settings.

Setting	Strategy	Costs per person ($)	QALYs per person	Incremental cost per person ($)	Incremental QALYs (95% CI)	Average ICURs (95% CI) ($)
Rural	No screening	214.07	12.10231	—	—	—
	Community screening ^*^	228.36	12.10573	14.29	0.00342 (0.00145 to 0.00596)	4178.51 (3859.33 to 5342.96)
	Telemedicine screening ^*^	235.30	12.11145	21.23	0.00914 (0.00782 to 0.01024)	2323.04 (1023.16 to 3902.51)
	Telemedicine screening ^†^	235.30	12.11145	6.93	0.00572 (0.00407 to 0.00715)	1211.93 (896.07 to 1590.31)

Urban	No screening	220.78	12.18423	—	—	—
	Community screening ^*^	238.59	12.18890	17.81	0.00467 (0.00309 to 0.00582)	3812.91 (2905.72 to 4167.39)
	Telemedicine screening ^*^	244.25	12.19386	23.47	0.00963 (0.00780 to 0.01174)	2437.28 (1241.60 to 3519.85)
	Telemedicine screening ^†^	244.25	12.19386	5.66	0.00496 (0.00326 to 0.00657)	1141.13 (859.03 to 1402.85)

^*^Compared with no screening. ^†^Compared with community screening. Costs are given in US dollars. QALY = quality-adjusted life year. ICUR = incremental cost-utility ratio.

By further comparison, compared to community screening programs, telemedicine screening yielded an ICUR of 1212 (95% CI 896 to 1590) per incremental QALY gained in rural setting and 1141 (95% CI 859 to 1403) in urban setting, which both meet the criterion for a significantly cost-effective health intervention (Table 3). Thus, telemedicine screening programs are believed to be dominant compared to community programs.

The one-way sensitivity analysis analyzed all model parameters and listed the parameters with a relatively substantial effect on the results. Varying the parameters within the tested range did not cause the ICUR to exceed the cost-utility threshold of three times per capita GDP in tornado diagrams. The most influential parameters were transition probability from treated PDR with photocoagulation to severe visual impairment, followed by the annual diagnosed PDR. Other parameters, including the community screening compliance, utility of PDR and DME, and screening cost, had moderate effects on the model outputs. The tornado diagram gives the factors with the most impact on the ICUR given different search strategies and settings (Supplementary 3). Through analysis, screening for DR was robust and insensitive to uncertainty for a wide range of variable values adjusted in our model by both community and telemedicine. In all of our strategies and settings, the screening programs showed clear benefits and were cost-effective: within one per capita GDP ($7000 in rural areas and $12,000 in urban areas).

ICURs were robust to randomly distributed parameters in all of our search strategies and settings in the probabilistic sensitivity analysis. The cost-effectiveness acceptability curve from the probabilistic sensitivity analysis shows the proportion of iterations, where both screening strategies in both rural and urban settings were cost-effective at the willingness-to-pay threshold of one time per capita GDP and three times per capita GDP (Supplementary 4). Under the threshold of one time per capita GDP, the telemedicine screening program dominated community screening in most of the simulations in rural (71.6%) and urban (80.9%) settings. Similarly, telemedicine screening programs dominated community screening in most simulations in rural (67.8%) and urban (78.9%) settings under a threshold of three times per capita GDP.

The model evaluated the costs and health rewards of the different screening intervals under different settings and strategies (Supplementary 5). Compared with longer screening intervals, if the ICURs were lower than the threshold of three times per capita GDP, the screening intervals were considered acceptable. A comparison shows that for community screening programs, screening every 3 years in rural settings and every 2 years under urban settings were best screening intervals. For telemedicine screening programs, screening every 2 years in both rural and urban settings was the best strategy. By further calculation, in rural settings, screening every 2 years by telemedicine yielded an ICUR of 3272 per QALY gained than every 3 years by community screening strategy. In urban settings, screening every 2 years by telemedicine dominated every 2 years by community screening strategy, with fewer costs and more QALY. Thus, we derived screening every 2 years by telemedicine was the best type of screening and interval combination both in rural and urban settings.

## 4. Discussion

To our knowledge, our study is the first to analyze the cost-utility of telemedicine compared with community screening programs for DR under rural and urban settings in China using a Markov model, from the societal perspective. The cost-utility analysis showed that both telemedicine and community screening for DR were cost-effective under rural and urban settings. The results suggest that it is economically reasonable to adopt telemedicine screening programs at the national primary care level. In the existing studies, telemedicine screening programs for DR have been proven cost-effective, although most studies were conducted in high-income nations [24]. In developing nations, Rachapelle et al. [37] reported that telemedicine screening programs are cost-effective for DM patients in rural India compared with no screening. Ben et al. [47] found that systematic teleophthalmology-based screening programs are cost-effective in Brazil compared to opportunistic ophthalmology-referral-based screening, with an ICUR of $4976/QALY for systematic teleophthalmology-based screening, which is under the ICUR threshold ($14,953/QALY). In China, DR screening is effective in patients newly diagnosed with T2 diabetes, and screening intervals for ≥4 years were cost-effective (ICUR<$7,485/QALY) [48]. In our study, we derived screening every 2 years by telemedicine was the best type of screening and interval combination both in rural and urban settings.

However, there are insufficient skilled retinal specialists and image graders to assess fundus images to satisfy current demand. In the future, the use of validated automated grading software based on AI will improve the cost-utility and cost-effectiveness of screening [49]. The development of deep learning has significantly improved in the detection of DR, providing a high sensitivity (87–90%) and specificity (98%) [50, 51]. Moreover, with the development of technology for telecommunication, cloud storage, miniaturization of equipment, and application of AI to the automatic interpretation of retinal images, there may be further optimization in productivity, quality assurance, and sustainability. However, the cost-utility of these new technologies should be evaluated before clinical implementation. As recently reported from an economic analysis modeling study in Singapore, an AI-assisted diagnosis model that combines a deep learning system with human assessment could save $15 per patient compared to the conventional model [52].

DR screening programs cannot be separated from diabetes management. Diabetes self-management education in the United States has made clear provisions for medication management, self-monitoring, and comprehensive evaluation [53]. However, domestic diabetes management started relatively late and remains in the preliminary stages. The gap between domestic and international diabetes management systems mainly lies in the following points: the management path of diagnosis and treatment is not standardized; the communication channel between doctors and patients is not fully open; the distribution of diabetes education in China is uneven. Subsequently, new diabetes and DR health management systems should be investigated. For instance, “Internet plus community” can be promoted to innovate the diabetes health management mode, with patients at the center to drive comprehensive, long-term, and systematic management. The data can be efficiently collected and transferred to physicians via digital systems. Likewise, retinal photos taken by fundus cameras or other portable devices can be sent to specialized referral centers.

**Figure 2:**
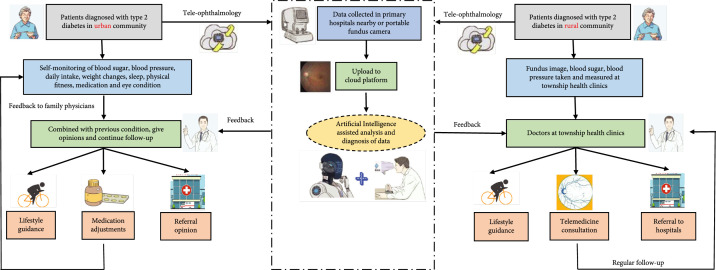
Workflow of diabetic retinopathy screening strategies in the management of diabetes in urban and rural communities. DM patients in urban communities can feedback their general health data and eye condition collected in primary hospitals and evaluated by cloud platforms to family or community physicians for further lifestyle guidance, medication adjustment, or referral opinion. For patients in rural communities, DM patients can take fundus images at local clinics and upload them to cloud platforms to obtain diagnosis and treatment suggestions from ophthalmologists. Combined with the blood glucose, blood pressure, and other systemic conditions, doctors at township health clinics will provide guidance and conduct telemedicine consultations or referrals to higher-level medical institutions when necessary. DM = diabetes mellitus.

Here, we refer to other studies on the integration of AI into DR screening programs and propose a new pattern of DR screening in the community management of diabetes that can be applied in the future (Figure 2) [54]. Recent cost-effectiveness and cost-utility analysis of semiautomated and fully automated DR screening program in developed countries has provided a strong economic rationale for deep learning systems as an assistive tool to screen for DR [55, 56]. Currently, community screening is applied in rural and remote areas more frequently, while telemedicine screening is mainly applied in urban areas in China. The application of AI in both screening settings can help improve the screening efficiency and promote the equity, accessibility, and acceptability of eye healthcare resources. In remote areas, the combination of AI and community screening models can help improve the efficiency and accuracy of screening; and in urban areas, the application of AI can help meet the demand of urban residents for more frequent screening and higher healthcare standards. Teleophthalmology is expected to thrive with the accurate integration of AI, combined with telecommunication tools such as smartphones, powerful hardware, advanced software, wireless devices, and remote video tools [57].

Moreover, prevention and control strategies for DR should be designed according to local situations in China, determined by local economic development and the quality of health services [2]. For urban communities where the prevalence of DM is relatively higher, teleophthalmology applied to community family health care can help improve compliance for follow-up by being faster and more convenient for medical consultations and reducing unnecessary excessive medical burdens caused by visiting tertiary hospitals. For rural communities where people have relatively low health-related knowledge and compliance with health-related behavior and there is a lack of access to health care, teleophthalmology patterns can provide care and consultation to patients in remote areas.

However, our study has some limitations. First, clinical treatment for DME is more complex, involving clinically significant macular edema, DME with PDR, and recurrent DME, whereas our model was more idealized for the setting of treatment cost and transition probability. Accurate screening for DME also depends on the use of optical coherence tomography (OCT), but the use of OCT has not been proven to be cost-effective as a first-line screening [58, 59]. Second, similar to other health economic studies, there is marked methodological heterogeneity, limitations, and data gaps, which reduce the comparability across different research results. We referred to utility values from studies in Singapore and India, while it would be more accurate and convincing if the health utility measurement in China was available. We also idealized that annualized cost for fix assets was calculated by assuming a life span 5 years and no salvage value. According to the Lancet Global Health Commission on Global Eye Health, more standardized data should be collected from diverse settings, which requires financial investment and the capacity for local data collection [60]. Finally, the Markov model here did not allow for the influence of recovery from a more severe condition to better health, and it may underestimate the benefits of the screening. There is a potential to improve cost-utility by differentiating low-risk and high-risk patients, which requires further evaluation. Subsequent evaluations can explore the best screening interval in depth for different age groups to formulate a more targeted policy.

## 5. Conclusions

In brief, both telemedicine and community screening for DR in rural and urban settings were cost-effective, and telemedicine screening programs can be more cost-effective than community screening in China from the societal perspective. The results from the models of this study are more likely applicable under other settings with low labor costs and a high prevalence of DR with low opportunistic detection rates, such as low-income and middle-income nations.

## Data Availability

All data relevant to the study are included in the article or supplementary materials.

## Abbreviations

DM: Diabetes mellitus
DME: Diabetic macular edema
DR: Diabetic retinopathy
VEGF: Vascular endothelial growth factor
ICUR: Incremental cost-utility ratio
NPDR: Nonproliferative diabetic retinopathy
PDR: Proliferative diabetic retinopathy
QALY: Quality-adjusted life years
DALY: Disability-adjusted life years
GDP: Gross domestic product
AI: Artificial intelligence
CE: Cost-effectiveness
OCT: Optical coherence tomography.

## Supplementary Materials

Supplementary Materials Supplementary 1. Care pathways for screening, referral and opportunistic detection. Supplementary 2. Variation range and distributions for parameters in our model Supplementary 3. Tornado diagram showing the extent to which uncertainty in the individual parameters affects the incremental cost-utility ratio (ICUR) by different screening strategies in rural and urban settings Supplementary 4. Cost-effectiveness (CE) acceptability curve showing the proportion of iterations in which the screening was cost-effective/dominant at each willingness-to-pay threshold Supplementary 5. Cost-utility of different diabetic retinopathy screening intervals Supplementary 6. Consolidated Health Economic Evaluation Reporting Standards (CHEERS) Checklist.
