# Supplementary material for: Cost-Utility Analysis of Screening for Diabetic Retinopathy in China
Source: Health Data Sci. 2022 Mar 12;2022:9832185. doi: 10.34133/2022/9832185 (PMC10904067; doi:10.34133/2022/9832185)
Supplement: Supplementary Materials — Supplementary 1. Care pathways for screening, referral and opportunistic detection. Supplementary 2. Variation range and distributions for parameters in our model Supplementary 3. Tornado diagram showing the extent to which uncertainty in the individual parameters affects the incremental cost-utility ratio (ICUR) by different screening strategies in rural and urban settings Supplementary 4. Cost-effectiveness (CE) acceptability curve showing the proportion of iterations in which the screening was cost-effective/dominant at each willingness-to-pay threshold Supplementary 5. Cost-utility of different diabetic retinopathy screening intervals Supplementary 6. Consolidated Health Economic Evaluation Reporting Standards (CHEERS) Checklist. [file 9832185.f1.docx]

Supplementary Online Content

Supplementary 1. Care pathways for screening, referral and opportunistic detection.

Supplementary 2. Variation range and distributions for parameters in our model

Supplementary 3. Tornado diagram showing the extent to which uncertainty in the individual parameters affects the incremental cost-utility ratio (ICUR) by different screening strategies in rural and urban settings

Supplementary 4. Cost-effectiveness (CE) acceptability curve showing the proportion of iterations in which the screening was cost-effective/dominant at each willingness-to-pay threshold

Supplementary 5. Cost-utility of different diabetic retinopathy screening intervals

Supplementary 6. Consolidated Health Economic Evaluation Reporting Standards (CHEERS) Checklist

This supplementary material has been provided by the authors to give readers additional information about our work.

Supplementary 1. Care pathways for screening, referral and opportunistic detection.


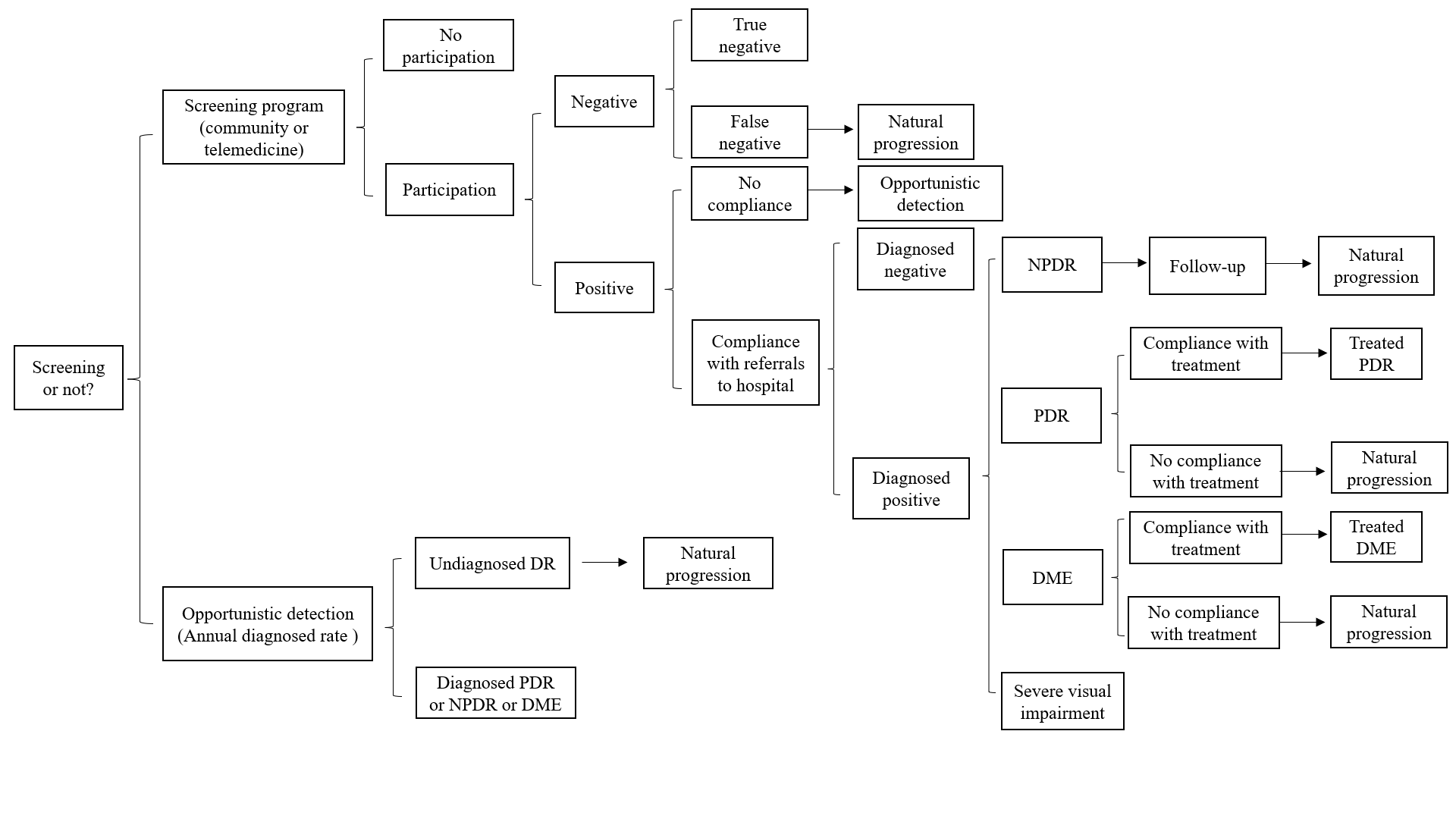


DR= diabetic retinopathy; NPDR= non-proliferative diabetic retinopathy; PDR= proliferative diabetic retinopathy; DME= diabetic macular edema.

Supplementary 2. Variation range and distributions for parameters in our model

| Parameters | Probability | Range for sensitivity analysis | | | Distributions used in the probability sensitivity analysis | Resource |
| --- | --- | --- | --- | --- | --- | --- |
|  |  | Low | High |  |  |  |
| Prevalence of NPDR (rural) | 0.256 | 0.181 | 0.348 |  | Beta (74.14,215.48) | ^1,2^ |
| Prevalence of PDR (rural) | 0.016 | 0.003 | 0.071 |  | Beta (98.38,6050.62) | ^1,2^ |
| Prevalence of DME (rural) | 0.035 | 0.032 | 0.039 | ±10% | Beta (96.47,2659.68) | ^3^ |
| Prevalence of SVI (rural) | 0.001 | 0.0009 | 0.0011 | ±10% | Beta (99.9,99799.1) | ^1,2^ |
| Annual diagnosed PDR (rural) | 0.350 | 0.315 | 0.385 | ±10% | Beta (64.65,120.06) | Unpublished data |
| Annual diagnosed DME (rural) | 0.540 | 0.486 | 0.594 | ±10% | Beta (45.46,38.73) | Unpublished data |
| Prevalence of NPDR (urban) | 0.149 | 0.106 | 0.204 |  | Beta (84.95,485.19) | ^1,2^ |
| Prevalence of PDR (urban) | 0.011 | 0.005 | 0.023 |  | Beta (98.89,8891.02) | ^1,2^ |
| Prevalence of DME (urban) | 0.026 | 0.023 | 0.029 | ±10% | Beta (97.37,3647.78) | ^3^ |
| Prevalence of SVI (urban) | 0.001 | 0.0009 | 0.0011 | ±10% | Beta (99.9,99799.1) | ^1,2^ |
| Annual diagnosed PDR (urban) | 0.420 | 0.378 | 0.462 | ±10% | Beta (57.58,79.52) | Unpublished data |
| Annual diagnosed DME (urban) | 0.650 | 0.585 | 0.715 | ±10% | Beta (34.35,18.5) | Unpublished data |
| Transition possibility from normal to NPDR | 0.0328 | 0.0184 | 0.0578 |  | Beta (96.69,2851.09) | ^4-8^ |
| Transition possibility from NPDR to PDR | 0.0024 | 0.0009 | 0.0063 |  | Beta (98.93,9234.03) | ^4-8^ |
| Transition possibility from NPDR to DME | 0.0107 | 0.0096 | 0.0118 | ±10% | Beta (98.92,9145.88) | ^4-8^ |
| Transition possibility from PDR to SVI | 0.0106 | 0.0040 | 0.0278 |  | Beta (97.19,3398.93) | ^4-8^ |
| Transition possibility from PDR to DME | 0.0290 | 0.0261 | 0.0319 | ±10% | Beta (97.07,3250.2) | ^4-8^ |
| Transition possibility from DME to SVI | 0.0500 | 0.0450 | 0.0550 | ±10% | Beta (94.95,1804.05) | ^4-8^ |
| Transition possibility from normal to PDR | 0.0278 | 0.0100 | 0.0744 |  | Beta (99.76,41465.91) | ^4-8^ |
| Transition possibility from treated PDR to SVI | 0.0116 | 0.0005 | 0.0268 |  | Beta (98.83,8420.86) | ^9^ |
| Transition possibility from treated DME to SVI | 0.0300 | 0.0270 | 0.0330 | ±10% | Beta (96.97,3135.36) | ^10^ |
| Utility of normal | 0.95 | 0.92 | 0.99 | ±10% | Beta (4.05,0.21) | ^11,12^ |
| Utility of NPDR | 0.79 | 0.71 | 0.87 | ±10% | Beta (20.21,5.37) | ^11,12^ |
| Utility of PDR | 0.70 | 0.63 | 0.77 | ±10% | Beta (29.3,12.56) | ^11,12^ |
| Utility of DME | 0.70 | 0.63 | 0.77 | ±10% | Beta (29.3,12.56) | ^11,12^ |
| Utility of SVI | 0.55 | 0.50 | 0.61 | ±10% | Beta (44.45,36.37) | ^11,12^ |
| Probability of normal called NPDR (community) | 0.05 | 0.04 | 0.06 | ±10% | Beta (94.95,1804.05) | ^11-13^ |
| Probability of normal called normal (community) | 0.95 | 0.86 | 1.00 | ±10% | Beta (4.05,0.21) | ^11-13^ |
| Probability of NPDR called normal (community) | 0.22 | 0.20 | 0.24 | ±10% | Beta (77.78,275.77) | ^11-13^ |
| Probability of PDR called NPDR (community) | 0.03 | 0.02 | 0.04 | ±10% | Beta (96.97,3135.36) | ^11-13^ |
| Probability of PDR called normal (community) | 0.02 | 0.01 | 0.03 | ±10% | Beta (97.98,4801.02) | ^11-13^ |
| Sensitivity of DME (community) | 0.82 | 0.74 | 0.90 | ±10% | Beta (17.18,3.77) | ^11-13^ |
| Specificity of DME (community) | 0.79 | 0.71 | 0.87 | ±10% | Beta (20.21,5.37) | ^11-13^ |
| Probability of normal called NPDR (telemedicine) | 0.04 | 0.03 | 0.05 | ±10% | Beta (95.96,2303.04) | ^12-15^ |
| Probability of normal called normal (telemedicine) | 0.96 | 0.86 | 1.00 | ±10% | Beta (3.04,0.13) | ^12-15^ |
| Probability of NPDR called normal (telemedicine) | 0.42 | 0.38 | 0.46 | ±10% | Beta (57.58,79.52) | ^12-15^ |
| Probability of PDR called NPDR (telemedicine) | 0.19 | 0.17 | 0.21 | ±10% | Beta (80.81,344.51) | ^12-15^ |
| Probability of PDR called normal (telemedicine) | 0.02 | 0.01 | 0.03 | ±10% | Beta (97.98,4801.02) | ^12-15^ |
| Sensitivity of DME (telemedicine) | 0.80 | 0.72 | 0.88 | ±10% | Beta (19.2,4.8) | ^12-15^ |
| Specificity of DME (telemedicine) | 0.95 | 0.86 | 1.00 | ±10% | Beta (4.05,0.21) | ^12-15^ |
| Compliance with hospital check (rural) | 0.18 | 0.16 | 0.20 | ±10% | Beta (81.82,372.74) | ^16^ |
| Compliance with hospital check (urban) | 0.23 | 0.21 | 0.25 | ±10% | Beta (76.77,257.01) | ^16^ |
| Compliance with treatment for PDR (rural) | 0.375 | 0.340 | 0.410 | ±10% | Beta (62.13,103.54) | Unpublished data |
| Compliance with treatment for DME (rural) | 0.245 | 0.221 | 0.270 | ±10% | Beta (75.26,231.91) | Unpublished data |
| Compliance with treatment for PDR (urban) | 0.545 | 0.49 | 0.60 | ±10% | Beta (44.96,37.53) | Unpublished data |
| Compliance with treatment for DME (urban) | 0.350 | 0.315 | 0.385 | ±10% | Beta (64.65,120.06) | Unpublished data |
| Compliance with community screening (rural) | 0.75 | 0.68 | 0.83 | ±10% | Beta (24.25,8.08) | Unpublished data |
| Compliance with telemedicine screening (rural) | 0.80 | 0.72 | 0.88 | ±10% | Beta (19.2,4.8) | Unpublished data |
| Compliance with community screening (urban) | 0.85 | 0.77 | 0.94 | ±10% | Beta (14.15,2.5) | Unpublished data |
| Compliance with telemedicine screening (urban) | 0.90 | 0.81 | 0.99 | ±10% | Beta (9.1,1.01) | Unpublished data |
| Screening cost (community) | 2.44 | 1.95 | 2.93 | ±20% | Gamma (16,0.15) | Local charge |
| Screening cost (telemedicine) | 1.84 | 1.47 | 2.21 | ±20% | Gamma (16,0.12) | Local charge |
| Hospital check cost | 100 | 80 | 120 | ±20% | Gamma (16,6.25) | Local charge |
| Treatment cost for PDR | 137.6 | 110.08 | 165.12 | ±20% | Gamma (16,8.6) | Local charge |
| Maintain cost for PDR/ DME | 122.32 | 97.86 | 146.78 | ±20% | Gamma (16,7.65) | Local charge |
| Treatment cost for DME | 1741.46 | 1393.17 | 2089.75 | ±20% | Gamma (16,108.84) | Local charge |
| Cost for SVI | 8800 | 4400 | 13200 | ±50% | Gamma (16,550) | ^17^ |

DR= diabetic retinopathy; NPDR= non-proliferative diabetic retinopathy; PDR= proliferative diabetic retinopathy; DME= diabetic macular edema; SVI= severe visual impairment

Reference:

1. Song P, Yu J, Chan KY, et al. Prevalence, risk factors and burden of diabetic retinopathy in China: a systematic review and meta-analysis. J Glob Health. 2018;8(1):010803.

2. Wang FH, Liang YB, Zhang F, et al. Prevalence of diabetic retinopathy in rural China: the Handan Eye Study. Ophthalmology. 2009; 116(3):461-467.

3. Xie XW, Xu L, Wang YX, et al. Prevalence and associated factors of diabetic retinopathy. The Beijing Eye Study 2006. Graefes Arch Clin Exp Ophthalmol. 2008; 246(11):1519-1526.

4. Sabanayagam C, Banu R, Chee ML, et al. Incidence and progression of diabetic retinopathy: a systematic review. Lancet Diabetes Endocrinol. 2019;7(2):140-149.

5. Xu J, Xu L, Wang YX, et al. Ten-year cumulative incidence of diabetic retinopathy. The Beijing Eye Study 2001/2011. PLoS One. 2014;9(10):e111320.

6. Lee R, Wong TY, Sabanayagam C. Epidemiology of diabetic retinopathy, diabetic macular edema and related vision loss. Eye Vis (Lond). 2015; 30(2): 17.

7. Moshfeghi A, Garmo V, Sheinson D, et al. Five-Year Patterns of Diabetic Retinopathy Progression in US Clinical Practice. Clin Ophthalmol. 2020; 29(14): 3651-3659.

8. Raman R, Ganesan S, Pal SS, et al. Incidence and Progression of Diabetic Retinopathy in Urban India: Sankara Nethralaya-Diabetic Retinopathy Epidemiology and Molecular Genetics Study (SN-DREAMS II), Report 1. Ophthalmic Epidemiol. 2017;24(5):294-302.

9. Evans JR, Michelessi M, Virgili G. Laser photocoagulation for proliferative diabetic retinopathy. Cochrane Database Syst Rev. 2014(11):CD011234.

10. Weiss M, Sim DA, Herold T, et al. COMPLIANCE AND ADHERENCE OF PATIENTS WITH DIABETIC MACULAR EDEMA TO INTRAVITREAL ANTI-VASCULAR ENDOTHELIAL GROWTH FACTOR THERAPY IN DAILY PRACTICE. Retina. 2018; 38(12): 2293-2300.

11. Nguyen HV, Tan GS, Tapp RJ, et al. Cost-effectiveness of a National Telemedicine Diabetic Retinopathy Screening Program in Singapore. Ophthalmology. 2016;123(12):2571-2580.

12. Wu B, Li J, Wu H. Strategies to Screen for Diabetic Retinopathy in Chinese Patients with Newly Diagnosed Type 2 Diabetes: A Cost-Effectiveness Analysis. Medicine (Baltimore). 2015;94(45):e1989.

13. Bragge P, Gruen RL, Chau M, et al. Screening for presence or absence of diabetic retinopathy: a meta-analysis. Arch Ophthalmol. 2011;129(4):435-444.

14. Whited JD. Accuracy and reliability of teleophthalmology for diagnosing diabetic retinopathy and macular edema: a review of the literature. Diabetes Technol Ther. 2006; 8(1): 102-111.

15. Shi L, Wu H, Dong J, et al. Telemedicine for detecting diabetic retinopathy: a systematic review and meta-analysis. Br J Ophthalmol. 2015;99(6):823-831.

16. Wang D, Ding X, He M, et al. Use of eye care services among diabetic patients in urban and rural China. Ophthalmology. 2010;117(9):1755-1762.

17. Tang J, Liang Y, O'Neill C, et al. Cost-effectiveness and cost-utility of population-based glaucoma screening in China: a decision-analytic Markov model. Lancet Glob Health. 2019;7(7):e968-e978.

Supplementary 3. Tornado diagram showing the extent to which uncertainty in the individual parameters affects the incremental cost-utility ratio (ICUR) by different screening strategies in rural and urban settings


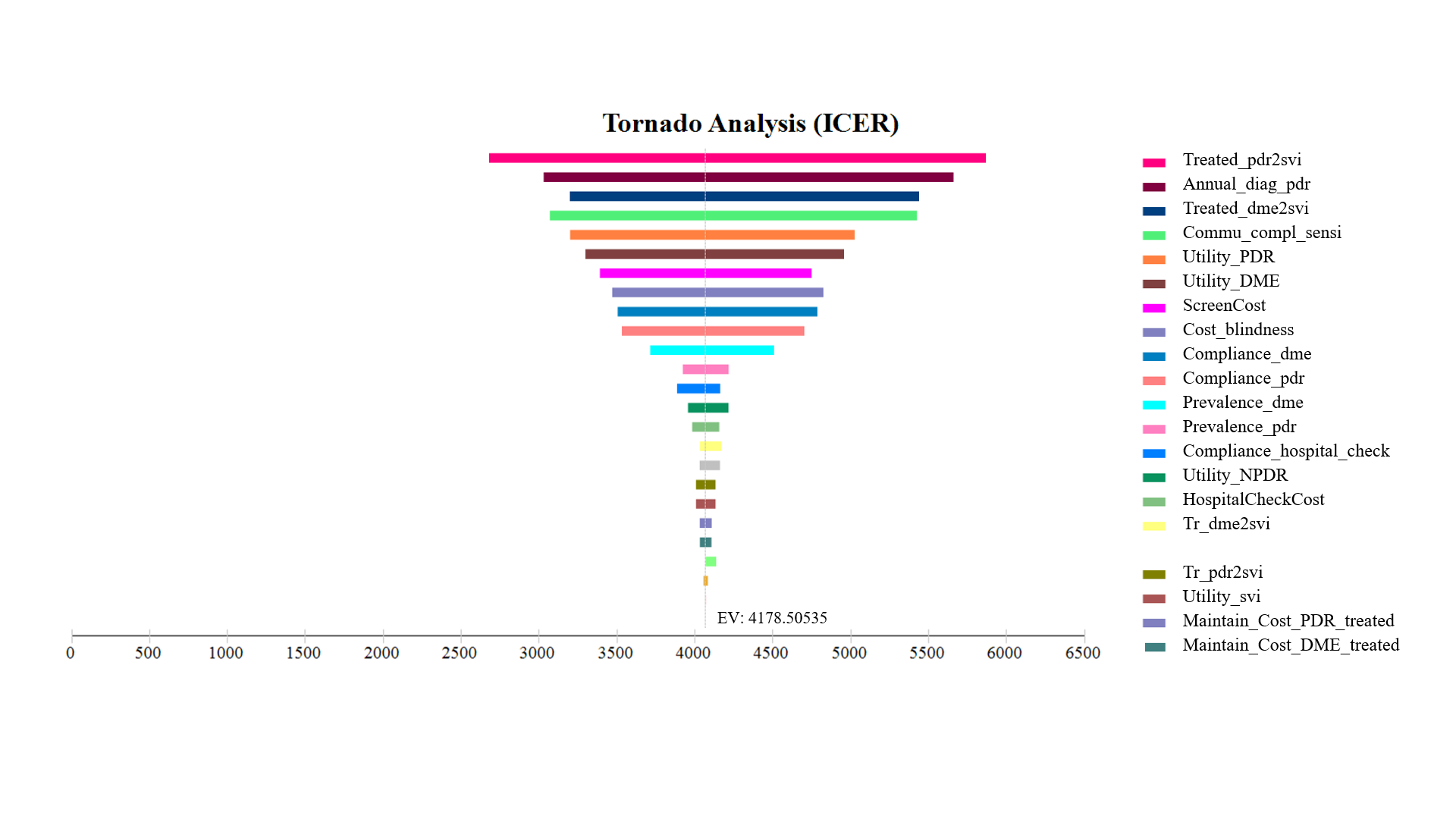


1. Community screening vs. No screening in rural setting


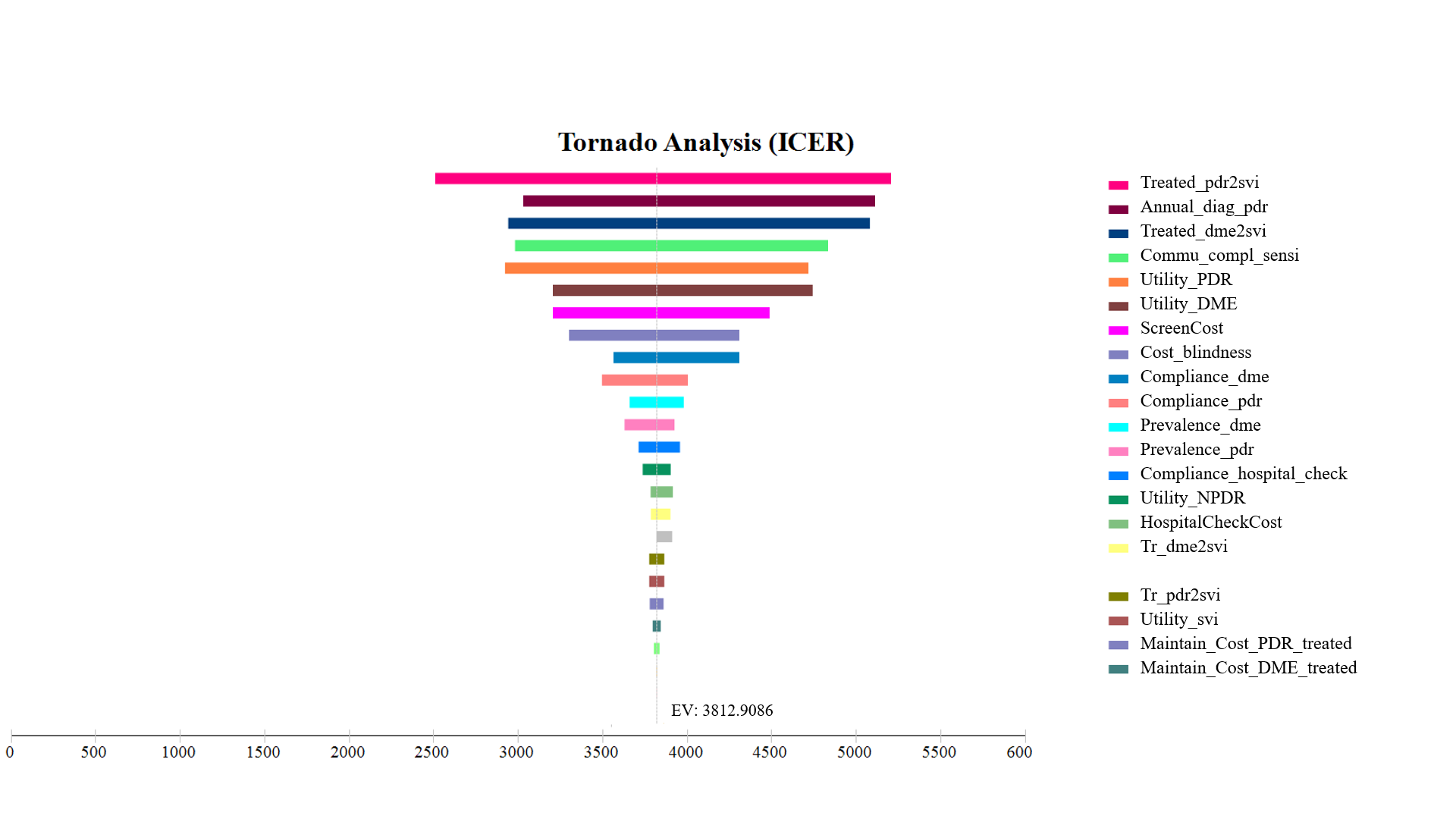


1. Community screening vs. No screening in urban setting


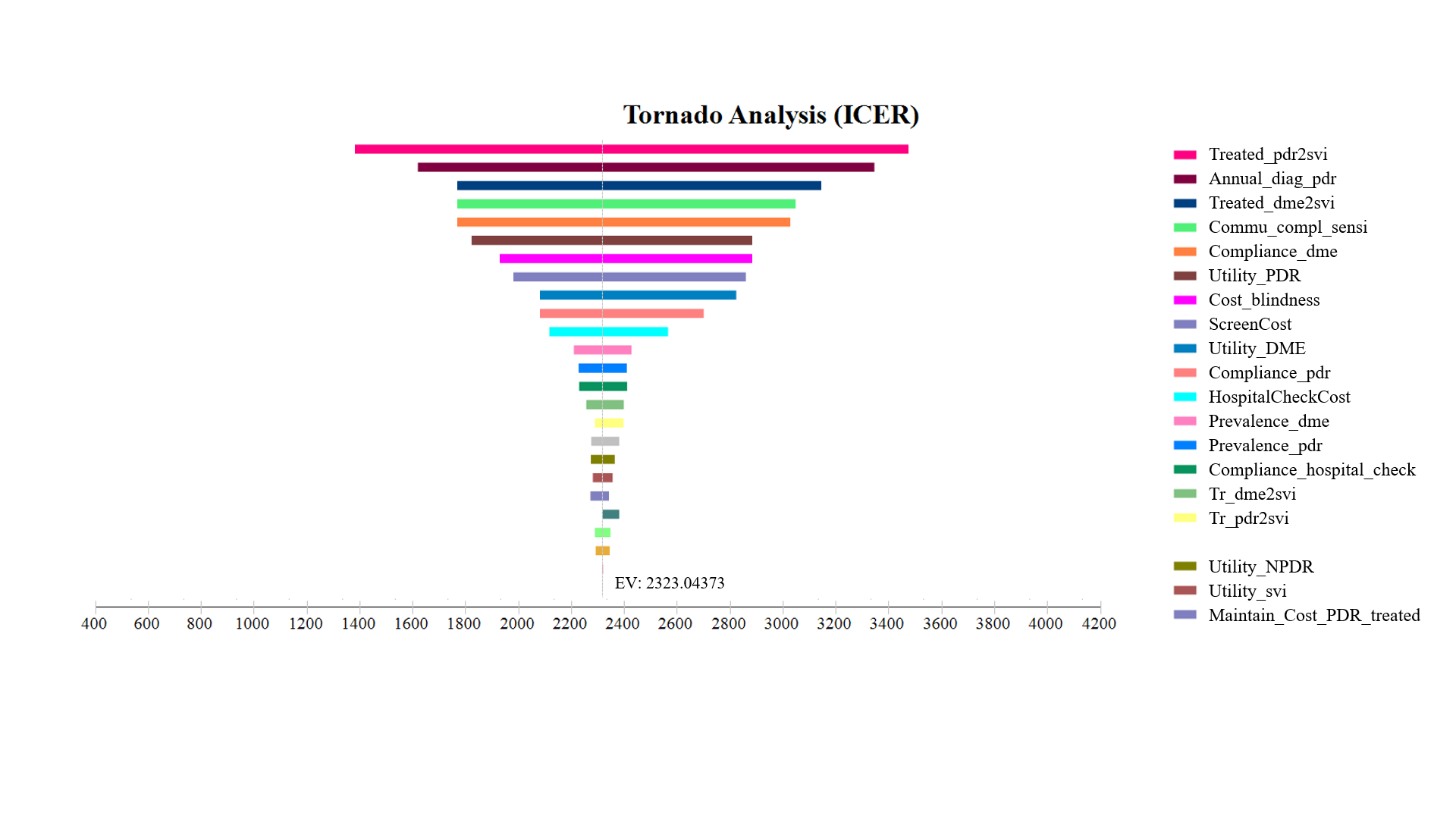


1. Telemedicine screening vs. No screening in rural setting


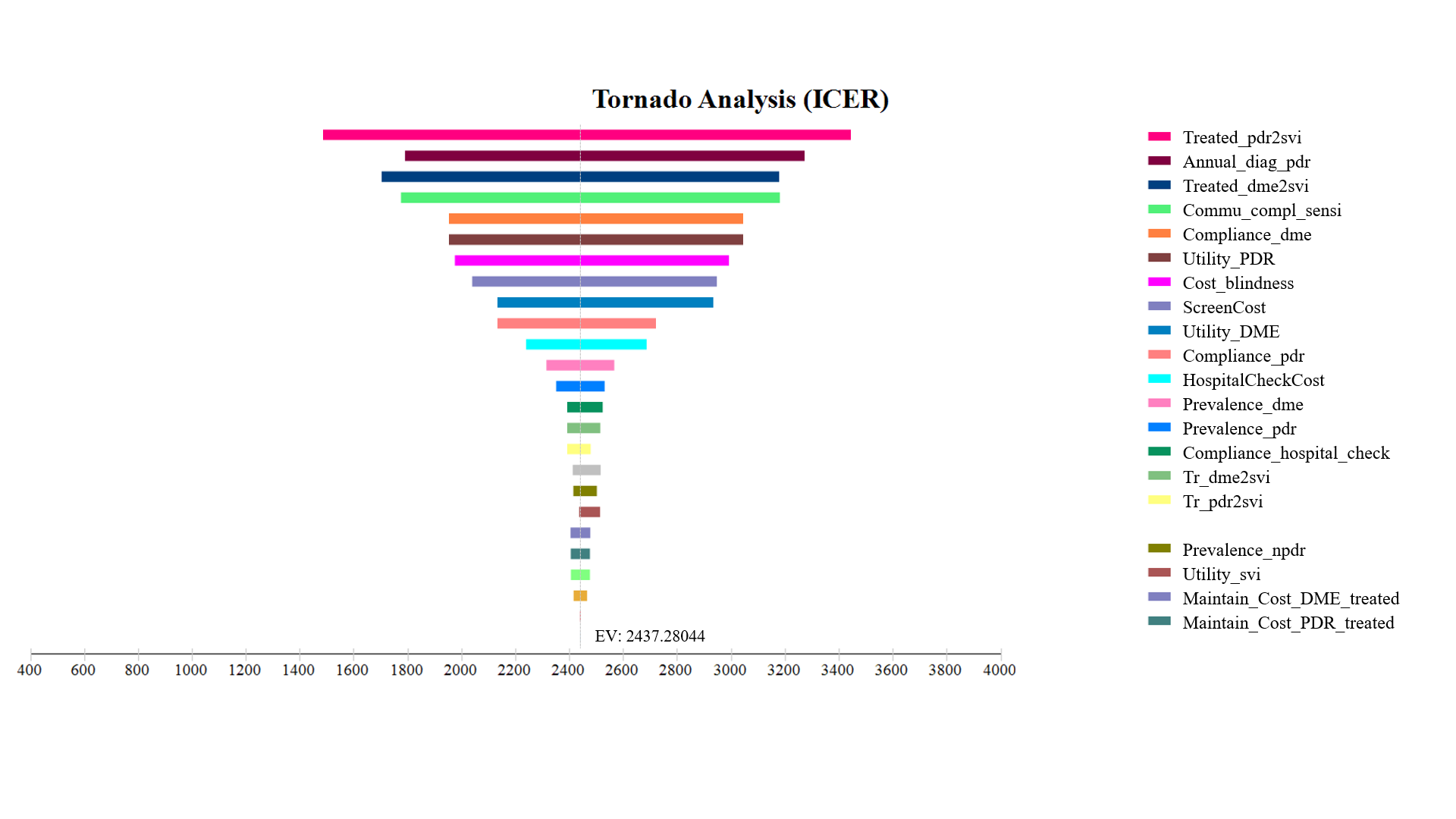


1. Telemedicine screening vs. No screening in urban setting


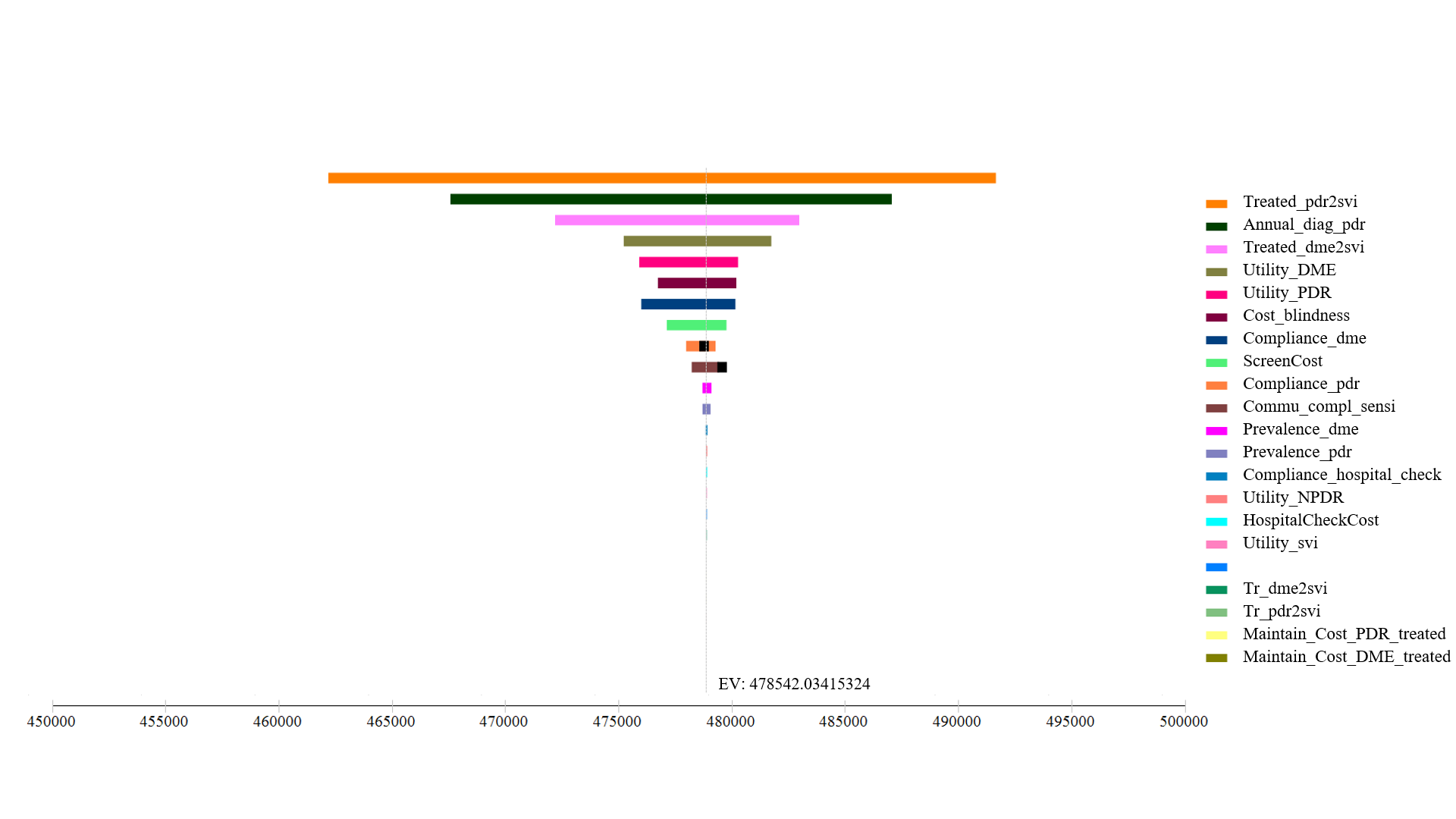


E. Telemedicine screening vs. Community screening in rural setting


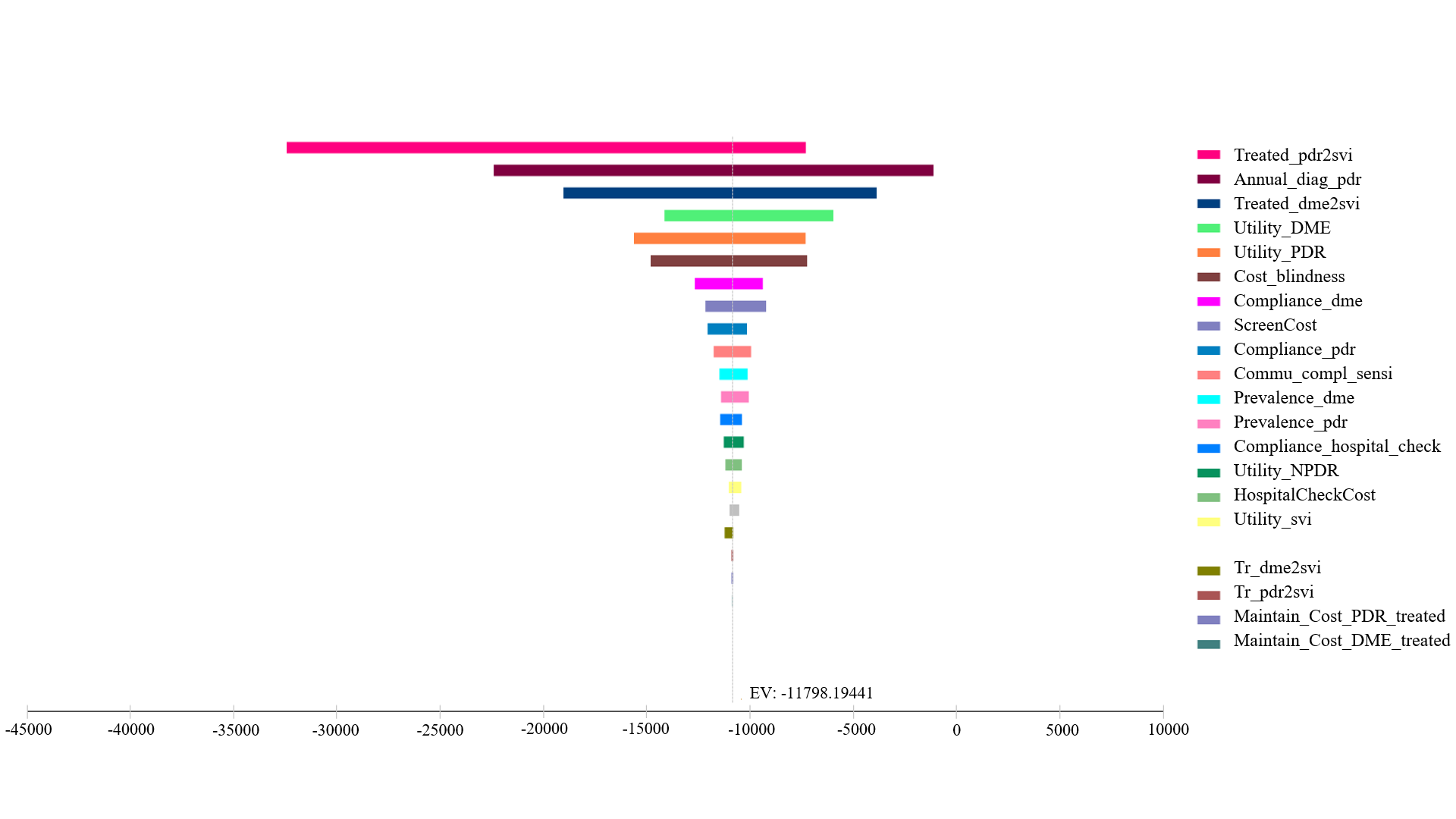


F. Telemedicine screening vs. Community screening in urban setting

Supplementary 4. Cost-effectiveness (CE) acceptability curve showing the proportion of iterations in which the screening was cost-effective/dominant at each willingness-to-pay threshold.


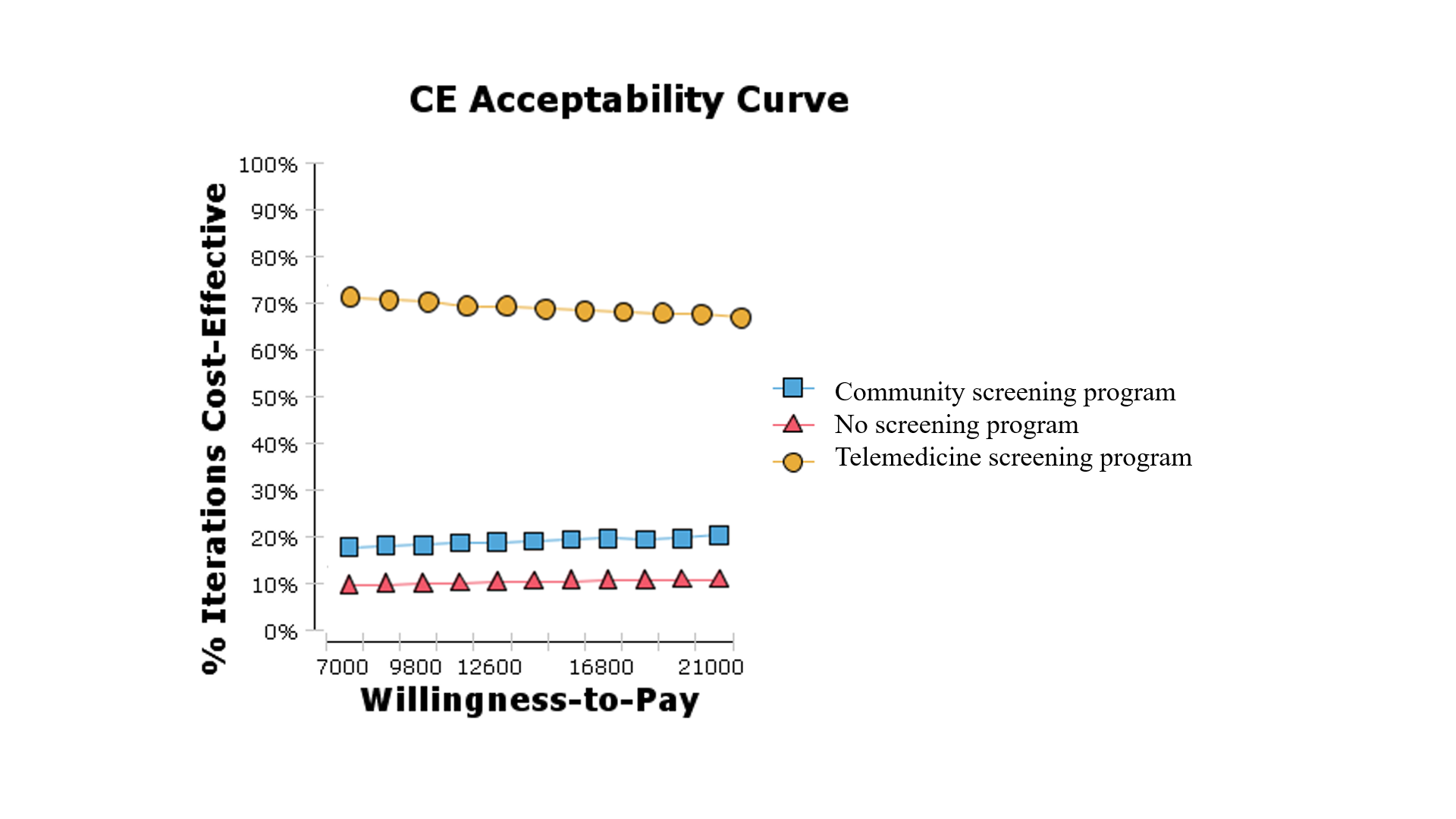


1. Community screening vs. Telemedicine screening vs. No screening program in rural setting


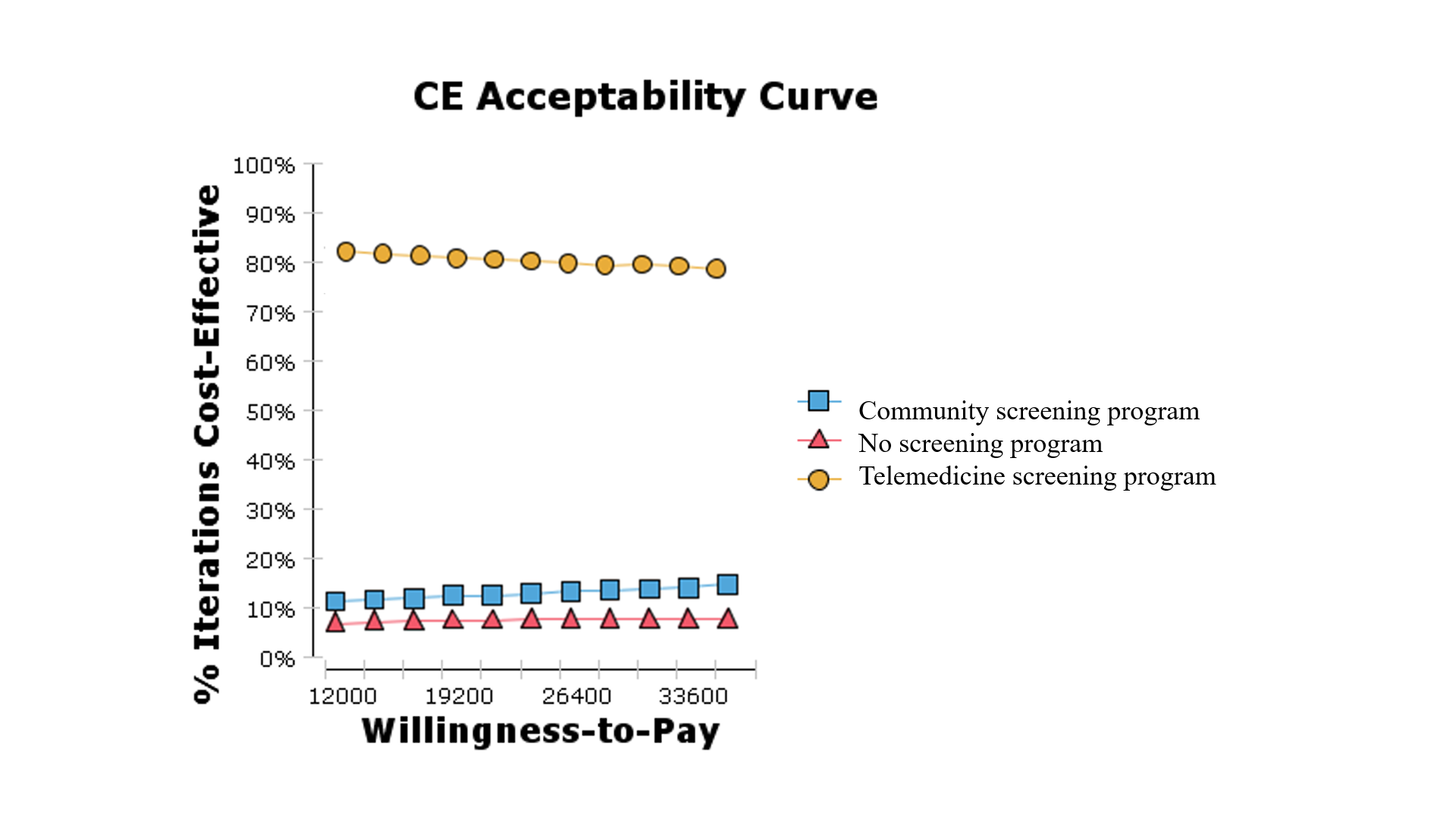


1. Community screening vs. Telemedicine screening vs. No screening program in urban setting

Supplementary 5. Cost-utility of different diabetic retinopathy screening intervals

| Strategy | Setting | Interval | QALYs per person | Costs per person, $ | ICURs, $ | Comparison screening interval for ICUR calculation |
| --- | --- | --- | --- | --- | --- | --- |
| Community Screening | Rural | Once-off | 12.10573 | 228.36 | - | - |
|  |  | Every 5 years | 12.10460 | 237.43 | 8023.16 | Once-off |
|  |  | Every 4 years | 12.10395 | 253.31 | 14019.52 | Every 5 years |
|  |  | **Every 3 years** | **12.10383** | **266.86** | **20264.78** | **Every 4 years** |
|  |  | Every 2 years | 12.10341 | 305.59 | 33287.09 | Every 3 years |
|  |  | Every 1 years | 12.10287 | 408.12 | 62852.45 | Every 2 years |
| Telemedicine Screening | Rural | Once-off | 12.11145 | 235.30 | - | - |
|  |  | Every 5 years | 12.10777 | 259.27 | 6513.89 | Once-off |
|  |  | Every 4 years | 12.10736 | 264.30 | 12280.43 | Every 5 years |
|  |  | Every 3 years | 12.10655 | 277.41 | 16185.70 | Every 4 years |
|  |  | **Every 2 years** | **12.10548** | **299.84** | **20961.38** | **Every 3 years** |
|  |  | Every 1 years | 12.10549 | 332.86 | 37103.62 | Every 2 years |
| Community Screening | Urban | Once-off | 12.18890 | 238.59 | - | - |
|  |  | Every 5 years | 12.18764 | 248.72 | 8043.63 | Once-off |
|  |  | Every 4 years | 12.18689 | 266.57 | 13920.15 | Every 5 years |
|  |  | Every 3 years | 12.18653 | 291.91 | 22496.21 | Every 4 years |
|  |  | **Every 2 years** | **12.18608** | **323.64** | **30158.36** | **Every 3 years** |
|  |  | Every 1 years | 12.18571 | 392.65 | 48293.45 | Every 2 years |
| Telemedicine Screening | Urban | Once-off | 12.19386 | 244.25 | - | - |
|  |  | Every 5 years | 12.19261 | 251.88 | 6103.56 | Once-off |
|  |  | Every 4 years | 12.19224 | 256.49 | 12456.12 | Every 5 years |
|  |  | Every 3 years | 12.19180 | 264.19 | 17495.80 | Every 4 years |
|  |  | **Every 2 years** | **12.19137** | **275.98** | **27432.39** | **Every 3 years** |
|  |  | Every 1 years | 12.19075 | 307.80 | 51324.61 | Every 2 years |

ICUR=incremental cost-utility ratio.

Supplementary 6. Consolidated Health Economic Evaluation Reporting Standards (CHEERS) Checklist

**Items to include when reporting economic evaluations of health interventions**

The **ISPOR CHEERS Task Force Report**, *Consolidated Health Economic Evaluation Reporting Standards (CHEERS)—Explanation and Elaboration: A Report of the ISPOR Health Economic Evaluations Publication Guidelines Good Reporting Practices Task Force*, provides examples and further discussion of the 24-item CHEERS Checklist and the CHEERS Statement. It may be accessed via the *Value in Health* or via the ISPOR Health Economic Evaluation Publication Guidelines – CHEERS: Good Reporting Practices webpage: <http://www.ispor.org/TaskForces/EconomicPubGuidelines.asp>

| **Section** | **Item No** | **Recommendation** | **Reported on page No/line No** |
| --- | --- | --- | --- |
| **Title and Abstract** | | | |
| Title | 1 | Identify the study as an economic evaluation or use more specific terms such as “cost-effectiveness analysis”, and describe the interventions compared. | **Page 1** |
| Abstract | 2 | Provide a structured summary of objectives, perspective, setting, methods (including study design and inputs), results (including base case and uncertainty analyses), and conclusions. | **Page 2** |
| **Introduction** | | | |
| Background and objectives | 3 | Provide an explicit statement of the broader context for the study.  Present the study question and its relevance for health policy or practice decisions. | **Page 3** |
| **Methods** | | | |
| Target population and subgroups | 4 | Describe characteristics of the base case population and subgroups analyzed, including why they were chosen. | **Page 4** |
| Setting and location | 5 | State relevant aspects of the system(s) in which the decision(s) need(s) to be made. | **Page 4-5** |
| Study perspective | 6 | Describe the perspective of the study and relate this to the costs being evaluated. | **Page 5** |
| Comparators | 7 | Describe the interventions or strategies being compared and state why they were chosen. | **Page 4-5** |
| Time horizon | 8 | State the time horizon(s) over which costs and consequences are being evaluated and say why appropriate. | **Page 4** |
| Discount rate | 9 | Report the choice of discount rate(s) used for costs and outcomes and say why appropriate. | **Page 4** |

| Choice of health out- comes | 10 | Describe what outcomes were used as the measure(s) of benefit in the evaluation and their relevance for the type of analysis performed. | **Page 6** |
| --- | --- | --- | --- |
| Measurement of effectiveness | 11a | *Single study-based estimates*: Describe fully the design features of the single effectiveness study and why the single study was a sufficient source of clinical effectiveness data. |  |
|  | 11b | *Synthesis-based estimates*: Describe fully the methods used for identification of included studies and synthesis of clinical effectiveness data. | **Page 6** |
| Measurement and valuation of preference-based outcomes | 12 | If applicable, describe the population and methods used to elicit preferences for outcomes. | **-** |
| Estimating resources and costs | 13a | *Single study-based economic evaluation*: Describe approaches used to estimate resource use associated with the alternative interventions. Describe primary or secondary re- search methods for valuing each resource item in terms of its unit cost. Describe any adjustments made to approximate to opportunity costs. |  |
|  | 13b | *Model-based economic evaluation*: Describe approaches and data sources used to estimate resource use associated with model health states. Describe primary or secondary research methods for valuing each resource item in terms of its unit cost. Describe any adjustments made to approximate to opportunity costs. | **Supplementary 2** |
| Currency, price date, and conversion | 14 | Report the dates of the estimated resource quantities and unit costs. Describe methods for adjusting estimated unit costs to the year of reported costs if necessary. Describe methods for converting costs into a common currency base and the ex- change rate. | **Page 5** |
| Choice of model | 15 | Describe and give reasons for the specific type of decision- analytical model used. Providing a figure to show model structure is strongly recommended. | **Figure 2** |
| Assumptions | 16 | Describe all structural or other assumptions underpinning the decision-analytical model. | **Page 6** |
| Analytical methods | 17 | Describe all analytical methods supporting the evaluation. This could include methods for dealing with skewed, missing, or censored data; extrapolation methods; methods for pooling data; approaches to validate or make adjustments (such as half cycle corrections) to a model; and methods for handling population heterogeneity and uncertainty. | **Page 6** |
| **Results** | | | |
| Study parameters | 18 | Report the values, ranges, references, and, if used, probability distributions for all parameters. Report reasons or sources for distributions used to represent uncertainty where appropriate. Providing a table to show the input values is strongly recommended. | **Supplementary 3** |
| Incremental costs and outcomes | 19 | For each intervention, report mean values for the main categories of estimated costs and outcomes of interest, as well as mean differences between the comparator groups. If applicable, report incremental cost-effectiveness ratios. | **Page 7-8** |
| Characterizing uncertainty | 20a | *Single study-based economic evaluation*: Describe the effects of sampling uncertainty for the estimated incremental cost and incremental effectiveness parameters, together with the impact of methodological assumptions (such as discount rate, study perspective). |  |
|  | 20b | *Model-based economic evaluation*: Describe the effects on the results of uncertainty for all input parameters, and uncertainty related to the structure of the model and assumptions. | **Page 7-8, Supplementary 4-5** |
| Characterizing heterogeneity | 21 | If applicable, report differences in costs, outcomes, or cost- effectiveness that can be explained by variations between subgroups of patients with different baseline characteristics or other observed variability in effects that are not reducible by more information. | **Page 7-8** |
| **Discussion** | | | |
| Study findings, limitations, generalizability, and current knowledge | 22 | Summarize key study findings and describe how they support the conclusions reached. Discuss limitations and the generalizability of the findings and how the findings fit with current knowledge. | **Page 8-11** |
| **Other** | | | |
| Source of funding | 23 | Describe how the study was funded and the role of the fun- der in the identification, design, conduct, and reporting of the analysis. Describe other non-monetary sources of support. | **Page 11** |
| Conflicts of interest | 24 | Describe any potential for conflict of interest of study contributors in accordance with journal policy. In the absence of a journal policy, we recommend authors comply with International Committee of Medical Journal Editors recommendations. | **Page 11** |

For consistency, the CHEERS Statement checklist format is based on the format of the CONSORT statement checklist.

The **ISPOR CHEERS Task Force Report** provides examples and further discussion of the 24-item CHEERS Checklist and the CHEERS Statement. It may be accessed via the *Value in Health* link or via the ISPOR Health Economic Evaluation Publication Guidelines – CHEERS: Good Reporting Practices webpage: <http://www.ispor.org/TaskForces/EconomicPubGuidelines.asp>

The citation for the CHEERS Task Force Report is:

Husereau D, Drummond M, Petrou S, et al. Consolidated health economic evaluation reporting standards (CHEERS)- Explanation and elaboration: A report of the ISPOR health economic evaluations publication guidelines good reporting practices task force. Value Health 2013; 16: 231-50.
